# Black Pepper *(Piper nigrum)* for Tobacco Withdrawal: A Case Report

**DOI:** 10.1155/2022/5908769

**Published:** 2022-12-09

**Authors:** Jeremy Weleff, Samyukta Dore, Akhil Anand, Brian S. Barnett

**Affiliations:** ^1^Department of Psychiatry and Psychology, Center for Behavioral Health, Neurological Institute, Cleveland Clinic, Cleveland, OH 44106, USA; ^2^Department of Psychiatry, Yale University School of Medicine, New Haven, Connecticut, USA; ^3^Cleveland Clinic Lerner College of Medicine at Case Western Reserve University, EC-10 Cleveland Clinic, 9501 Euclid Ave, Cleveland, OH 44195, USA

## Abstract

Tobacco use continues to be one of humanity's most significant public health concerns, causing more than 8-million deaths annually. Existing treatments for tobacco use disorder are limited in efficacy and there is a strong need for identifying effective novel treatments. Small clinical trials indicate that black pepper *(Piper nigrum)* essential oil may be helpful for treating nicotine withdrawal and craving. However, we are unaware of any cases reporting the use of black pepper for these purposes in nonresearch settings. Here we present the case of a patient who inhaled combusted black pepper to self-medicate nicotine withdrawal when lacking access to tobacco cigarettes while incarcerated. Based on our patient's report, inhalation of combusted black pepper may have alleviated his tobacco withdrawal and cravings by reducing his automatic motor urge to smoke, quelling withdrawal-associated anxiety, and mimicking the sensorimotor experience of smoking tobacco cigarettes. Notably, our patient reported that inhalation of combusted black pepper for treatment of nicotine craving and withdrawal was common in his correctional facility. Though combusted black pepper is highly unlikely to be an appealing treatment outside of a correctional setting, this case suggests that further investigation of vaporized black pepper essential oil for tobacco cessation may be warranted.

## 1. Introduction

Despite a growing societal focus on mortality from opioids, stimulants, and novel psychoactive substances, tobacco use remains one of the deadliest public health concerns, killing more than 8 million people annually [1]. While existing tobacco cessation treatments such as varenicline, nicotine replacement therapy, and advice from clinicians are effective for many patients [2], there remains a strong need for development of novel tobacco cessation treatments. Novel treatments that reduce the intensity of tobacco withdrawal may be particularly helpful, since many patients struggle to overcome withdrawal symptoms in order to move on to early remission [3]. According to the *Diagnostic and Statistical Manual of Mental Disorders, Fifth Edition*, tobacco withdrawal occurs within 24 hours of cessation of or reduction in daily use of tobacco that has lasted for several weeks. It consists of four or more of the following signs or symptoms: irritability, frustration, or anger; anxiety; difficulty concentrating; increased appetite; restlessness; depressed mood; and insomnia [4].

The commonly used spice black pepper *(Piper nigrum)* has previously demonstrated antimicrobial, antioxidant, anticancer, antidiabetic, hypolipidemic, anti-inflammatory, analgesic, anticonvulsant, and neuroprotective effects [5]. Such studies have primarily been *in vitro* in nature. However, black pepper has been investigated in humans as a potential treatment for tobacco withdrawal, with two small clinical trials of vaporized black pepper essential oil producing promising results [6, 7]. The first of these studies, published in 1994, compared the effects of vaporized black pepper essential oil, vaporized menthol/mint, and unflavored air on tobacco smoking withdrawal symptoms, habit withdrawal symptoms (the sensation of holding a cigarette in one's mouth or hands), anxiety, satisfaction, perceived nicotine delivery, and lung function. Cigarette cravings were significantly reduced in the black pepper condition compared to control conditions [7]. In a 2013 study, participants reported larger reductions in nicotine craving after inhaling vaporized black pepper essential oil than after inhaling angelica essential oil [6]. Some companies now market black pepper aromatherapy products as potential treatments for tobacco use disorder and cravings and cite these studies as evidence of efficacy [8]; though there are currently no U.S. Food and Drug Administration (FDA) approved black pepper smoking cessation products.

Interestingly, it seems that knowledge of black pepper's potential role in treating tobacco withdrawal may have reached incarcerated populations. Here, we present what we believe to be the first published case of an individual combusting black pepper in condiment form to self-treat tobacco withdrawal.

## 2. Case

Our patient was a 40-year-old male with schizoaffective disorder depressive type, cannabis use disorder in sustained remission, and tobacco use disorder admitted for inpatient psychiatric treatment of suicidal ideation and psychosis. He was diagnosed with schizoaffective disorder at age 14. Upon arrival to our unit, he reported depressed mood over the prior few weeks in the context of social stressors and had developed suicidal ideation with a plan to overdose on his medications. A few days prior to admission, he had intentionally burned his hand. The patient had three previous psychiatric admissions, all following suicide attempts. He was first psychiatrically hospitalized at age 15, and his most recent psychiatric hospitalization was three years prior to admission for a suicide attempt by walking into traffic during a psychotic episode. He also struggled with chronic auditory hallucinations and paranoid ideation towards others. He had never received electroconvulsive therapy. On day five of his seven day admission, the patient's Patient Health Questionnaire-9 (PHQ-9) score was 9 (mild depression), General Anxiety Disorder-7 (GAD-7) was 7 (mild anxiety), and Community Assessment of Psychic Experiences-15 (CAPE-15) was 22 with a frequency score of 1.47 (ultrahigh risk for psychosis). His medications on admission were benztropine 2 mg nightly, fluoxetine 20 mg, fluphenazine 40 mg nightly, and haloperidol 20 mg nightly. He reported smoking 1-2 packs of cigarettes daily and chewing tobacco daily.

The patient reported that he was incarcerated in southern Ohio from 1996-2016 after assaulting several people with a baseball bat during a psychotic episode. During his incarceration he typically smoked 40 self-rolled tobacco cigarettes daily, but when unable to access tobacco due to lack of funds, he would smoke cigarettes crafted from cigarette rolling paper and two packets of black pepper condiment. He learned this strategy for addressing his tobacco withdrawal symptoms (including tobacco craving, poor sleep, anxiety, irritability, restlessness, and depressed mood) from other inmates. Though the combusted black pepper was harsh on his airways, frequently leading to long coughing fits, the patient would smoke up to four black pepper cigarettes per day when unable to obtain tobacco. He estimated that he smoked black pepper during approximately 30 episodes of nicotine withdrawal during his incarceration. He denied any long-term adverse health effects related to this behavior and denied cravings to smoke black pepper again. He denied experiencing any euphoria from the black pepper and stated that his primary use of it was “just to have some smoke hit my lungs since I didn't have tobacco”. He never smoked black pepper when tobacco was available, though he also denied any desire or intentional attempts to discontinue his tobacco use during his incarceration. The patient denied harboring any negative associations with black pepper after using it in this manner, and he continued to enjoy black pepper as part of his diet.

During his hospitalization, no chest imaging was conducted to assess for any carcinogenic or respiratory impact of the patient's tobacco use. However, a chest radiograph approximately six months prior to admission demonstrated no radiographic abnormalities. While hospitalized, the patient's fluoxetine was increased to target his depressed mood. He also expressed interest in smoking cessation and agreed to a trial of varenicline, which he tolerated well. His discharge medication regimen was benztropine 2 mg nightly, fluoxetine 40 mg daily, fluphenazine 40 mg nightly, haloperidol 20 mg nightly, and varenicline 1 mg twice daily. The patient has not been seen in our hospital system since discharge, so we are unable to comment on the long-term efficacy of varenicline for his tobacco use disorder.

## 3. Discussion

While two previous studies have investigated the use of vaporized black pepper essential oil for tobacco withdrawal [6, 7], we believe this to be the first published report of an individual combusting black pepper in condiment form to treat tobacco withdrawal. For our patient, this unusual self-treatment strategy appears to have been at least partially effective for tobacco withdrawal and did not produce compulsive use of its own. Unfortunately, employing black pepper in this manner did not produce long-term tobacco abstinence. Notably, our patient used black pepper specifically for treatment of tobacco withdrawal, so it is unknown whether black pepper might have aided him in achieving long term abstinence from tobacco had that been his goal.

Our patient reported three potential mechanisms by which inhaled combusted black pepper might reduce tobacco withdrawal symptoms. The first of these was a reduction in his impulsive, automatic urge to smoke tobacco. Automatic urges, as opposed to need or pleasure urges, were the most dominant types of smoking urges reported by patients seeking smoking cessation services in one study [9]. In that study, higher levels of automatic urges predicted failure to sustain abstinence following a smoking cessation attempt, while there was no association between abstinence and levels of need or pleasure urges. The second potential mechanism was a possible reduction in withdrawal-associated anxiety. Studies in mice have demonstrated anti-anxiolytic, mood-elevating, and cognitive-enhancing effects of black pepper and its primary alkaloid piperine, possibly by attenuation of oxidative stress in the amygdala [10, 11]. The third potential mechanism was the production of a sensorimotor experience similar to that of smoking tobacco cigarettes. Not only did our patient's black pepper cigarettes appear similar to tobacco cigarettes, but they also produced both a pungent taste and a crackling sound similar to those produced by combusted tobacco cigarettes.

Our patient also reported that the black pepper smoke produced sensations in the chest and respiratory tract similar to those produced by tobacco, a phenomenon reported by participants in a previous black pepper aromatherapy trial [7]. The reinforcing effects of airway stimulation from puffing cigarettes are well established and are an ongoing area of interest in novel smoking cessation treatment development [12–14]. In a 2020 study investigating respiratory sensations and sensory-related smoking cues associated with e-cigarette use among people who failed to quit smoking combustible tobacco cigarettes via traditional medications but were able to do so using e-cigarettes [15], 91% of participants believed the sensations of e-cigarette use contributed to their cessation of combustible tobacco cigarettes.

Additionally, though our patient did not mention this specifically, smoking black pepper in cigarette form also allowed him to continue the repetitive hand-to-mouth movements thought to be an important component of addiction to tobacco cigarette smoking [16]. This may have also played a role in alleviation of his tobacco cravings. When considering who might possibly benefit from use of black pepper for tobacco cessation, it is important to note that our patient had schizoaffective disorder. Individuals with psychotic disorders have a much higher prevalence of nicotine use than the general population [17], possibly due to improvement in their negative and cognitive symptoms by nicotine [18]. Increasing evidence suggests nicotine may have a causal relationship with psychosis [19], and tobacco use disorder in patients with psychotic disorders is often quite resistant to current treatment modalities [20, 21]. As a result, tobacco-related deaths are responsible for approximately 53% of deaths in patients with schizophrenia [22]. Whether our patient's schizoaffective disorder played a role in the apparent efficacy of combusted black pepper for his tobacco withdrawal or lack of efficacy for long-term tobacco cessation is unknown. Our case also highlights the potential for identifying unique strategies employed by prisoners with substance use disorders for treating withdrawal and the need to learn more about the role of substance use and treatment of substance use disorders in “prison culture” [23].

According to our patient, use of combusted black pepper for treatment of tobacco withdrawal was widespread in his prison. If such a practice was indeed widespread, it prompts the question of how it became so prevalent. One possibility is that the efficaciousness of utilizing black pepper as an alternative to tobacco when faced with nicotine withdrawal prompted its widespread use. While directly smoking black pepper as was reported by the patient likely produces harsh smoke and toxic chemicals, the potential to develop a safer vaporized black pepper alternative with the same benefits for tobacco withdrawal remains promising. This case study suggests that vaporized essential black pepper oil may warrant further investigation as a smoking cessation aid.
